# Accuracy and impact on quality of life of real-time continuous glucose monitoring in children with hyperinsulinaemic hypoglycaemia

**DOI:** 10.3389/fendo.2023.1265076

**Published:** 2023-09-26

**Authors:** Madhini Sivasubramanian, Parizad Avari, Clare Gilbert, Louise Doodson, Kate Morgan, Nick Oliver, Pratik Shah

**Affiliations:** ^1^Department of Paediatric Endocrinology, Great Ormond Street Hospital for Children NHS Foundation Trust, London, United Kingdom; ^2^University College London, Institute of Child Health, London, United Kingdom; ^3^Faculty of Health and Wellbeing, University of Sunderland in London, London, United Kingdom; ^4^Department of Metabolism, Digestion and Reproduction, Imperial College London, London, United Kingdom; ^5^Department of Paediatric Endocrinology, The Royal London Children’s Hospital, Barts Health NHS Trust, London, United Kingdom

**Keywords:** hyperinsulinism, continuous glucose monitoring, hypoglycaemia, neurodevelopment, time below range

## Abstract

**Objective:**

Continuous glucose monitoring (CGM) is the standard of care for glucose monitoring in children with diabetes, however there are limited data reporting their use in hyperinsulinaemic hypoglycaemia (HH). Here, we evaluate CGM accuracy and its impact on quality of life in children with HH.

**Methods:**

Real-time CGM (Dexcom G5 and G6) was used in children with HH aged 0-16years. Data from self-monitoring capillary blood glucose (CBG) and CGM were collected over a period of up to 28days and analysed. Quality of life was assessed by the PedsQL4.0 general module and PedsQL2.0 family impact module, completed by children and their parents/carers before and after CGM insertion. Analysis of accuracy metrics included mean absolute relative difference (MARD) and proportion of CGM values within 15, 20, and 30% or 15, 20, and 30 mg/dL of reference glucose values >100 mg/dL or ≤100 mg/dL, respectively (% 15/15, % 20/20, % 30/30). Clinical reliability was assessed with Clarke error grid (CEG) analyses.

**Results:**

Prospective longitudinal study with data analysed from 40 children. The overall MARD between reference glucose and paired CGM values (n=4,928) was 13.0% (Dexcom G5 12.8%, Dexcom G6 13.1%). The proportion of readings meeting %15/15 and %20/20 were 77.3% and 86.4%, respectively, with CEG analysis demonstrating 97.4% of all values in zones A and B. Within the hypoglycaemia range (<70 mg/dL), the median ARD was 11.4% with a sensitivity and specificity of 64.2% and 91.3%, respectively. Overall PedsQL child report at baseline and endpoint were 57.6 (50.5 – 75.8) and 87.0 (82.9 – 91.2), and for parents were 60.3 (44.8 – 66.0) and 85.3 (83.7 – 91.3), respectively (both p<0.001).

**Conclusion:**

Use of CGM for children with HH is feasible, with clinically acceptable accuracy, particularly in the hypoglycaemic range. Quality of life measures demonstrate significant improvement after CGM use. These data are important to explore use of CGM in disease indications, including neonatal and paediatric diabetes, cystic fibrosis and glycogen storage disorders.

## Highlights

- There has been limited research into the accuracy of real-time continuous glucose monitoring (CGM) in children at high risk of hypoglycaemia due to hyperinsulinaemia.
- This is the largest dataset reporting the accuracy of real-time continuous glucose monitoring in this cohort, with results demonstrating clinically acceptable accuracy, including in the hypoglycaemia range.
- Furthermore, use of CGM is associated with significant improvement in quality of life in children and their parents.
- These data are important for the use of CGM in children with rare disease indications, such as those with hyperinsulinism or rare metabolic disorders.

## Introduction

Hyperinsulinaemic hypoglycaemia (HH) is caused by dysregulation of insulin secretion from pancreatic β-cells (1). It is the main cause of persistent hypoglycaemia in neonates and infants, putting them at significant risk of permanent brain damage and even sudden death (2). Children with HH are at high risk of neurological deficit with hypoglycaemic episodes (3). The long-term effects of neonatal and childhood hypoglycaemia include an impact on visual-motor integration, motor skills, and academic attainment (4). Neonatal hypoglycaemia is associated with a two-to-three-fold increased risk of specific cognitive deficits in early childhood (2-5years), and general cognitive impairment and literacy and numeracy problems in later childhood (6-11years) (5). In 60 children with hyperinsulinism followed for a period of 5 years, 46.7% of them had at least one form of neurodevelopmental delay (6).

The care and management of children with HH can be complex (7). Regular capillary blood glucose measurement (CBG) by heel or finger prick is the standard of care to monitor blood glucose concentration in the hospital as well as at home, supporting the identification and management of hypoglycaemic episodes. However, blood glucose levels may need to be measured often, and up to every 10-15 minutes at the time of hypoglycaemia, especially where children are unable to communicate the symptoms of hypoglycaemia.

Continuous glucose monitoring (CGM) provides information on real-time glucose concentration, the rate and direction of change, and alerts and alarms for predicted, or established, hypoglycaemia below customisable thresholds (8). CGM is used in children with type 1 diabetes and is supported by a robust evidence base in paediatric and adult type 1 diabetes care (9, 10). CGM may be a useful monitoring tool to identify, or even predict, hypoglycaemia in HH, with potential to reduce the burden of CBG monitoring and the discomfort and pain associated with it. Moreover, CGM may enable identification of otherwise undetected episodes of hypoglycaemia especially those with asymptomatic hypoglycaemia and may have the potential to reduce the risk of neuroglycopenia in the developing brain.

There is limited evidence supporting CGM accuracy in the low blood glucose range in babies and young children, and little data supporting CGM use in children with HH (11). Previous studies in children with HH showed that CGM (Dexcom G4-G6) tends to under read compared with CBG measurements (12, 13). In another study, CGM (Dexcom G6) demonstrated an over reading compared to CBG (14), similar to another study with Freestyle Libre 1 (15). However, CGM can be used to help to understand glycaemic patterns and support prevention of severe hypoglycaemia (16).

In order to explore the potential for CGM to attenuate exposure to hypoglycaemia in children with HH, and thereby possibly reducing the risk of neurodevelopmental delay, data supporting the feasibility and accuracy of CGM in this population are required. The aim of this study is to determine the accuracy of real-time CGM in children with HH and to report pilot quality of life outcomes associated with CGM use.

## Methods

### Study design and participants

This is a prospective longitudinal study in children with HH conducted in both inpatient and home environment settings. Dexcom (San Diego, California) G5 and G6 CGM systems were used. The inclusion criteria were children with a diagnosis of HH within the age range of term babies with corrected gestational age >37weeks up to 16 years of age. Recruited neonates were over 2kg. The Dexcom G6 CGM system was used in children aged above 2 years. For children aged between 0-2 years, the Dexcom G5 system was used, as the G5 sensor has a manual inserter which enables the angle and force of the sensor insertion to be adjusted for smaller participants.

Parents and carers were requested to do at least six CBG readings by heel or finger prick sampling every 24 hours (fasting, pre-meals and before bedtime), and were supported to continue usual hypoglycaemia management during the duration of the study, based on CBG results. CBG was assessed using an ISO 15197:2013 compliant CBG device in line with the manufacturers’ instructions (Accu-Chek Inform II in an inpatient setting or with Accu-Chek Performa Nano glucometers at home). If the the CGM glucose was reported to be <72mmol/L (<4 mmol/L), the participant, carer or parent checked CBG concentration. If the initial capillary blood glucose was <63mg/dL (3.5mmol/L), a repeat sample was taken immediately from another site as per standard of care in children with HH. If the second value was <63mg/dL (3.5mmol/L), then this was treated as hypoglycaemia. All capillary blood glucose values along with CGM value were documented in a diary.

Parents and carers were taught how to insert and remove the sensor, and written information with emergency contact details were given to parents. Sensors for Dexcom G5 were changed every 7 days and Dexcom G6 every 10 days. Parents of children using the Dexcom G5 (<2 years old) were required to calibrate every 12 hours as per manufacturer guidance. For users of Dexcom G6, there was no requirement to calibrate the sensor.

Participants remained in the study for a period of 1 to 4 weeks. Glucose data were downloaded from all devices at the end of the study period. CBG readings were paired to the nearest 5 minute CGM value (either before or after). Ethical approval was granted by the London Fulham NHS Research and Ethics Committee.

### Quality of life study questionnaires

Age-appropriate and validated quality of life questionnaires (PedsQL version 2.0 for parents of participants aged 2-4 years, and PedsQL version 4.0 to all participants >4 years of age) were completed by participants and their families at the start and end of the monitoring period.

The PedsQL Generic Core Scales have child self-reporting forms designed for ages 5–7 (young child), 8–12 (child), and 13–18 (adolescent) years. Children >8 years report how much of a problem each item has been for them during the past one month. PedsQL assessments consists of 4 subscales on physical, emotional, social and school functioning, and include a Likert response scale (0 = never a problem; 1 = almost never a problem; 2 = sometimes a problem; 3 = often a problem; 4 = almost always a problem) for each item in each scale. A simplified, 3-point rating scale was used for younger children (0 = not at all; 2 = sometimes; 4 = a lot). Items were reverse-scored and transformed to a 0–100 scale where higher scores indicate better health related quality of life (i.e. 0 = 100, 1 = 75, 2 = 50, 3 = 25, 4 = 0). Scale scores were computed as the sum of the items divided by the number of items answered (this accounts for missing data). If greater than 50% of the items in the scale were missing, the scale score was not computed. A detailed description of scoring can be found via the PedsQL™ website (17).

### Primary and secondary outcomes

The primary endpoint was the overall mean absolute relative difference (MARD; %) between CGM compared to the reference CBG glucose. Secondary endpoints included median absolute relative difference (median ARD, %) for CGM and in the predefined glycaemic ranges: <70 mg/dL (<3.9 mmol/L); <63 mg/dL (<3.5 mmol/L); 70-180 mg/dL (3.9-10 mmol/L); >180 mg/dL (>10 mmol/L). The UK consensus in the management of hypoglycaemia in people with congenital hyperinsulinaemia is 63 mg/dL (3.5 mmol/L) (14).

Additional endpoint measures include Clarke error grid analysis, as well as percentage (%) time in range (TIR; 70–180 mg/dL; 3.9-10 mmol/L), % time in hypoglycaemia (<70 mg/dL; <3.9 mmol/L), and hyperglycaemia (>180 mg/dL; >10 mmol/L) calculated for the total study period.

### Statistical methods and data analysis

Glucose CGM sensor performance was evaluated by the absolute relative difference determined as an aggregate value from the total number of paired points. The performance evaluation also included the proportion of the CGM system values that are within ±20% of relative difference of reference value at glucose levels > 100 mg/dL (>5.6 mmol/L) and ±20 mg/dL (± 1.1 mmol/L) of absolute difference at glucose level ≤ 100 mg/dL (≤ 5.6 mmol/L), hereafter referred to as % 20/20, as well the proportion of the CGM system values that are within ± 15% of relative difference of reference value at glucose levels >100 mg/dL (>5.6mmol/L) and ±15 mg/dL (± 0.8 mmol/L) of absolute difference at glucose level ≤ 100 mg/dL (≤ 5.6 mmol/L), hereafter referred to as % 15/15. Sensitivity and specificity was also calculated for each hypoglycaemic threshold of <70mg/dL (<3.9mmol/L), <63mg/dL (<3.5mmol/L) and <54mg/dL (<3.0mmol/L), as well as the positive predictive value and negative predictive value.

Clarke error grid (CEG) analyses (18) were used to quantify the clinical accuracy of the CGM sensors, with Bland–Altman plots used to depict the data distribution and bias between sensor and the reference glucose. Measures of glycaemic variability were computed using EasyGV (v10.0) software (19).

Data have been presented as mean (standard deviation) and median (interquartile range [IQR]), unless otherwise stated. Distribution of data were assessed by quantile-quantile and density plots indicating non-normal distribution. Thus, the median ARDs have been reported alongside the MARD. Statistical tests were two-tailed and results considered statistically significant if p<0.05. Statistical analysis was performed using Stata version 15 (StataCorp, College Station, TX).

## Results

The study was conducted from June 2019 to March 2020 in a Paediatric Endocrine centre in London. There were 44 participants diagnosed with HH recruited in the study. Four participants were withdrawn from the study; one child developed mild skin rash at the sensor site and one developing swelling at the sensor insertion site. Two other participants did not submit CBG data to compare and were therefore withdrawn. Data from 40 children ranging from 0-16 years were included in the analysis.

There were 35 children aged >2 years old using Dexcom G6 and 5 children aged <2 years old using Dexcom G5. Twenty-seven (67.5%) children had a genetic cause identified, with remaining 13 (32.5%) having an unknown cause of HH. Baseline characteristics for the participants are summarized in **Table 1**. The mean sensor wear time for all participants was 21.2 ± 7.6 days.

**Table 1:** Baseline demographics for recruited participants (n=40).

Characteristics of the participants	Dexcom G6 (n=35)	Dexcom G5 (n=5)	Combined (n=40)
Age			
0-2 years	–	5 (100.0)	5 (100.0)
2-4 years	13 (37.1)	–	13 (37.1)
5-7 years	14 (40.0)	–	14 (40.0)
8-12 years	6 (17.1)	–	6 (17.1)
13-16 years	2 (5.7)	–	2 (5.7)
Gender			
Male Female	17 (48.6) 18 (51.4)	2 (40.0) 3 (60.0)	19 (47.5) 21 (52.5)
Diagnosis			
HI (unknown)	13 (37.4)	–	13 (32.5)
HI ABCC8	12 (34.3)	1 (20.0)	13 (32.5)
HI PMM2	2 (5.7)	–	2 (5.0)
HI GLUD1	2 (5.7)	1 (20.0)	3 (7.5)
HI HNF4α	2 (5.7)	1 (20.0)	3 (7.5)
HI Beckwith Wiedeman Syndrome	1 (2.9)	1 (20.0)	2 (5.0)
HI Fanconi Syndrome	1 (2.9)	–	1 (2.5)
HI Kabuki Syndrome	1 (2.9)	–	1 (2.5)
HI Von Willebrand disease Type	1 (2.9)	–	1 (2.5)
HI ZC4H2 Wieacker-Wiff syndrome	–	1 (20.0)	1 (2.5)
Treatment			
Diazoxide	8 (22.9)	1 (20.0)	9 (22.5)
Diazoxide ± other (i.e. octreotide/nifedipine)	11 (31.4)	1 (20.0)	12 (30.0)
Lanreotide	9 (25.8)	1 (20.0)	10 (33.3)
Octreotide	6 (17.1)	1 (20.0)	7 (17.5)
Not on any medication	1 (2.9)	1 (20.0)	2 (5.0)
Feeds			
Oral Feeds	28 (80.0)	3 (60.0)	31 (77.5)
Oral feeds and Gastric feeds	7 (20.0)	2 (40.0)	9 (22.5)

Results are expressed as median (IQR) or n (%). ABCC8, ATP Binding Cassette Subfamily C Member-8; GLUD1, glutamate dehydrogenase-1; HI, hyperinsulinism; HNF4α, hepatocyte nuclear factor-4α; PMM2, phosphomannomutase-2; ZC4H2, Zinc finger C4H2‐type containing gene.

### CGM accuracy

There were 4,928 CGM data points with paired CBG values (4,074 for Dexcom G6 and 854 for Dexcom G5). The MARD between paired CGM and CBG values was 13.0% (13.1% for Dexcom G6 and 12.8% for Dexcom G5; **Table 2**). For CBG values <3.9mmol/L (n= 779), the median ARD was 11.4% (11.7% for Dexcom G6 [n=599] and 10.5% for Dexcom G5 [n=180]). For clinically relevant hypoglycaemia (<63mg/dL; <3.5mmol/L) for participants with HH, the median ARD was 14.3% (11.6% for Dexcom G6 [n=288] and 14.3% for Dexcom G5 [n=74]). The overall %15/15 and %20/20 agreement was 77.3% and 86.4%, respectively (Dexcom G6: 77.1% and 86.3% respectively; Dexcom G5: 77.9% and 86.9% respectively).

**Table 2:** Sensitivity and specificity for varying glycaemic threshold using Dexcom G5 (n=854), G6 (n=4,074) and the combined dataset (n=4,928).

	Dexcom G6	Dexcom G5	Combined
*Measures of accuracy*			
**n paired data points**	4074	854	4928
**Mean ARD (%)**	13.1 (± 13.6)	12.8 ( ± 12.5)	13.0 (± 13.4)
**Median ARD (%)**	9.3 (4.8 – 17.0)	9.4 (4.0 – 17.0)	9.3 (4.8 – 17.0)
**Median ARD (%) <63mg/dL (<3.5mmol/L)**	14.3 (7.3 – 23.5)	11.6 (5.8 – 21.1)	14.3 (7.0– 23.3)
**Median ARD (%) <70mg/dL (<3.9mmol/L)**	11.7 (5.8 – 21.7)	10.5 (3.4 – 19.7)	11.4 (5.6 – 21.2)
**Median ARD (%) 70 -180mg/dL (3.9-10mmol/L)**	8.7 (4.4 – 15.2)	9.1 (4.0 – 16.1)	9.1 (4.5 – 16.3)
**Median ARD (%) >180 mg/dL (>10 mmol/L)**	6.0 (3.9 – 9.1)	8.7 (8.2 – 24.5)	6.1 (3.9 – 9.2)
**%15/15 (%)**	77.1	77.9	77.3
**%20/20 (%)**	86.3	86.9	86.4
*Glycaemic outcomes*			
**Times in range, %** <54mg/dL (<3.0mmol/L) <63mg/dL (<3.5mmol/L) <70mg/dL (<3.9mmol/L) 70 -180mg/dL (3.9-10mmol/L) >180mg/dL (>10mmol/L) >270mg/dL(>15mmol/L)	0.2 (0.0-0.6) 0.9 (0.2-2.1) 2.6 (0.6-5.9) 94.5 (86.9-96.8) 1.2 (0.5-5.6) 0.2 (0.0-0.6)	1.0 (0.8-4.9) 4.7 (3.6-13.5) 14.5 (11.5-21.4) 83.8 (76.9-88.5) 1.3 (0.7-1.3) 0.1 (0.0-0.1)	0.2 (0.0-0.8) 1.0 (0.2-3.2) 2.9 (1.2-7.5) 92.9 (85.5-96.6) 1.3 (0.5-4.0) 0.0 (0.0-0.2)
**Average blood glucose, mmol/L**	6.1 (5.8-6.7)	5.0 (4.9-5.6)	6.0 (5.6-6.7)
**CV, %**	23.2 (19.2-28.2)	27.5 (26.1-33.7)	24.1 (19.2-29.0)
**MAG**	2.4 (2.2-2.9)	2.4 (2.4-2.8)	2.4 (2.2-2.9)

Results are expressed as median (IQR)

**Figure 1:**
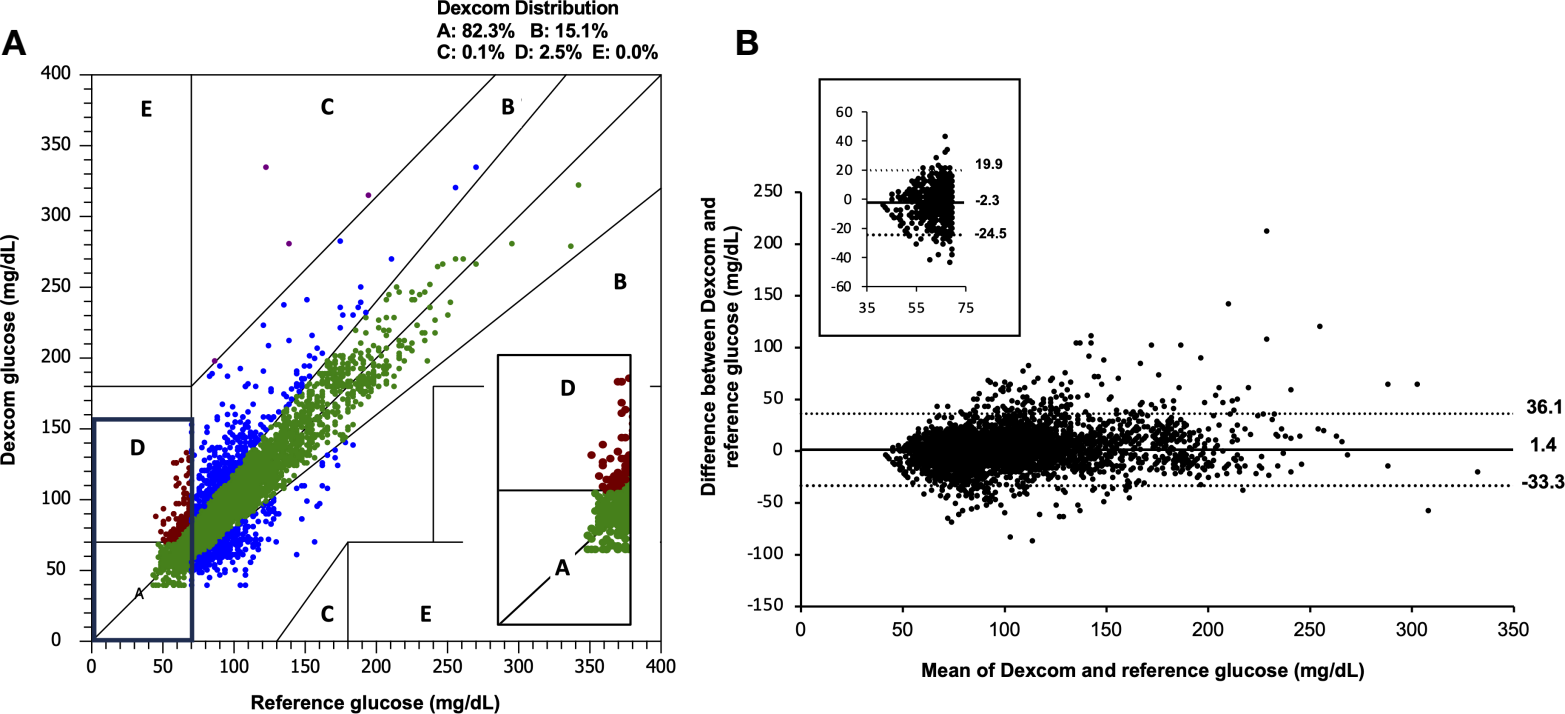
**(A)** Clarke error grid and **(B)** Bland Altmann analysis for combined Dexcom data (n=4,928) compared with capillary blood glucose. Inset images demonstrate the range of values observed when reference and sensor values are <70 mg/dL (3.9 mmol/L). The overall mean bias for Dexcom G6 was +1.4 mg/dL (+0.08mmol/L) and 95% limits of agreement were -33.3mg/dL (-1.85mmol/L), +36.1 mg/dL (2.01mmol/L). CBG, capillary blood glucose.

On the Clarke error grid analysis, 82.3% of points fell in the clinically accurate zone A, with 97.4% of all points falling in zones A and B (**Figure 1A**). Clarke error grids for Dexcom G5 and G6 are available as **Supplementary Material**. As illustrated on the Bland Altman analyses (**Figure 1B**), mean bias was +1.4mg/dL (+0.08mmol/L), with 95% limits of agreement for CGM to reference glucose CBG were -33.3mg/dL (-1.85mmol/L) and +36.1 mg/dL (+2.01mmol/L).

For the combined sensor data, sensitivity and specificity for hypoglycaemia (<63 mg/dL; <3.5 mmol/L) were 49.2% and 94.9% respectively, and 40.7% and 98.2% for hypoglycaemia (<54 mg/dL; <3.0mmol/L). When the hypoglycaemia threshold was increased to 70 mg/dL (3.9 mmol/L), sensitivity and specificity were 64.2% and 91.3% respectively. For the hypoglycaemia threshold of 70mg/dL (3.9mmol/L), the positive predictive value was 55% and the negative predictive value was 90% (**Table 3**).

**Table 3:** Measures of accuracy and glycaemic outcomes for Dexcom G5, G6 and the combined dataset.

	Dexcom G6 (n=4,074)		Dexcom G5 (n= 854)		Combined (n=4,928)	
Hypoglycaemia threshold	Sensitivity	Specificity	Sensitivity	Specificity	Sensitivity	Specificity
**<70mg/dL (<3.9mmol/L)**	61.9	92.3	70.4	82.8	64.2	91.3
**<63mg/dL (<3.5mmol/L)**	47.2	95.7	56.8	91.0	49.2	94.9
**<54mg/dL (<3.0mmol/L)**	40.0	98.6	43.8	96.7	40.7	98.2

Accuracy was assessed by matched CGM data points with paired CBG values (Dexcom G5 n=854; Dexcom G6 n=4,074 and the combined dataset n=4,928). Glycaemic outcomes assessed for children using Dexcom G5 (n=5), G6 (n=35) and the combined dataset (n=40). Results are expressed as median (IQR)/n (%) except where indicated. ARD, absolute relative difference; CGM, continuous glucose monitoring; CV, coefficient of variance; MAG, mean amplitude glucose.

### Times in glycaemic range and variability

Times in glycaemic range for children with HH are demonstrated in **Table 2**. The median (IQR) %TIR 70-180 mg/dL (3.9 – 10 mmol/L) for Dexcom G6 was 94.5 (86.9-96.8)% and Dexcom G5 was 83.8 (76.9-88.5)%. Percentage time below range <70 mg/dL (<3.9 mmol/L) was 2.6 (0.6-5.9)% for Dexcom G6 and 14.5 (11.5-21.4)% for Dexcom G5. For clinically relevant hypoglycaemia in HH, the percentage time below range <63mg/dL (<3.5mmol/L) for Dexcom G6 and G5 was 0.9 (0.2-2.1)% and 4.7 (3.6-13.5), respectively. Percentage time above range 180 mg/dL (>10 mmol/L) was 1.2 (0.5-5.6)% and 1.3 (0.7-1.3)% for Dexcom G6 and G5, respectively. The coefficient of variance was 23.2 (19.2-28.2)% and 27.5 (26.1-33.7)% for Dexcom G6 and G5, respectively.

### Quality of life outcomes

Quality of life questionnaires were available for 22 children aged >5 years old and family impact reports for 30 participants; remaining questionnaires were incomplete. Overall child reported PedsQL scores at baseline and after CGM use were 57.6 (50.5 – 75.8) and 87.0 (82.9 – 91.2) respectively, for all age groups (p<0.001; **Table 4**). Subgroup analyses for child reported PedsQL scores at baseline and endpoint were 56.5 (48.9 – 69.0) and 90.0 (86.5 – 91.3) for 5 to 7 years old (n=14; p=0.001); 75.5 (59.7 – 81.8) and 83.2 (76.1 – 83.7) for 8 to 12 years old (n=6; p=0.046); 63.0 (56.5 – 69.7) and 88.0 (87.0 – 89.1) for 13 to 16 years old (n=2).

**Table 4:** Quality of life outcomes pre- and post- CGM PedsQL report from children and their families.

Pre and Post CGM insertion PedsQL	N	Baseline (Pre-CGM)	End-point (Post-CGM)	Median change from baseline to endpoint	P value
**Family Impact Parent Report**	**30**	**45.4 (27.4 – 58.0)**	**80.6 (76.8 – 84.8)**	**+30.6 (17.9 – 46.1)**	**<0.001**
**Overall Child Report PedsQL 5-16yrs**	**22**	**57.6 (50.5 – 75.8)**	**87.0 (82.9 – 91.2)**	**+32.7 (10.1 – 38.6)**	**<0.001**
Child Report PedsQL 5-7yrs	14	56.5 (48.9 – 69.0)	90.0 (86.5 – 91.3)	+37.0 (28.6 – 41.2)	0.001
Child Report PedsQL 8-12yrs	6	75.5 (59.7 – 81.8)	83.2 (76.1 – 83.7)	+8.2 (4.3 – 14.3)	0.046
Teenager Report PedsQL 13-16yrs	2	63.0 (56.5 – 69.7)	88.0 (87.0 – 89.1)	+25.1 (19.6 – 30.4)	NA
**Overall Parent Report PedsQL 5-16yrs**	**22**	**60.3 (44.8 – 66.0)**	**85.3 (83.7 – 91.3)**	**+27.1 (20.2 – 41.0)**	**<0.001**
Parent Report PedsQL 5-7yrs	14	55.4 (46.5 – 63.7)	83.7 (82.9 – 90.5)	+30.4 (22.3 – 40.0)	0.001
Parent Report PedsQL 8-12yrs	6	70.1 (41.6 – 79.1)	88.0 (84.2 – 92.1)	+21.7 (7.3 – 42.7)	0.028
Parent Report PedsQL 13-16yrs	2	54.9 (49.7 – 60.1)	86.4 (86.1 – 86.7)	+31.5 (26.1 – 40.0)	NA

Results are expressed as median (IQR)/n (%). p<0.05 determines significance. Statistics not performed for teenagers aged 13-16 years old as only n=2. Text in bold are overall PedsQL scores.

Overall parent reported scores at baseline and after CGM use were 60.3 (44.8 – 66.0) and 85.3 (83.7 – 91.3) respectively, for children of all age groups (p<0.001). Family impact (parent reported) scores were 45.4 (27.4 – 58.0) and 80.6 (76.8 – 84.8) at baseline and endpoint, respectively (p<0.001).

### Safety outcomes

No serious device-related adverse events occurred during the study, apart from one child who developed mild skin rash at the sensor site and one child developed swelling at the sensor insertion site.

## Discussion

This is the largest study demonstrating accuracy and feasibility of real-time CGM for children with hyperinsulinaemic hypoglycaemia, including in the hypoglycaemic range. Furthermore, use of real-time CGM has a beneficial impact on the quality of life for children and their parents/carers.

The combined accuracy of all paired data points demonstrated an overall MARD of 13.0% across all clinically relevant glucose ranges, including the hypoglycaemic range. The MARD is slightly higher compared to that observed in accuracy studies in children with diabetes with Dexcom G5 and G6 (10% and 9%, respectively), likely due to more sensor data in this study being in the low and low-normal glucose range. On CEG analysis, 97.4% of values lie within zones A and B, meeting the established criteria that >95% of readings lie in either zone A or B (20). There was no systematic bias in the difference between CGM and CBG levels. Our sensor data are in keeping with previously reported results for hypoglycaemia sensitivity in CHI, which reportedly range from 43-73% (12–14).

Evidence from people with diabetes suggests that CGM is effective for people at high risk of hypoglycaemic events (21, 22), including people with impaired awareness of hypoglycaemia and recent severe hypoglycaemia requiring the assistance of a third party to treat (23, 24). Time spent in the hypoglycaemic range is consistently reduced with CGM use and the incidence of severe hypoglycaemia may be reduced (23, 25). Transferring these benefits to children with HH has potential to reduce the burden of CBG self-monitoring, reduce exposure to hypoglycaemia, including reducing the incidence of seizures associated with hypoglycaemia, and may prevent the deleterious effects of neuroglycopaenia.

The accuracy of CGM seen in the study population of children with HH is acceptable, suggesting that widespread use of CGM for children with HH has potential to be a safe, effective management tool especially in those children with asymptomatic hypoglycaemia and severe disease needing frequent monitoring. CGM may be particularly beneficial for young children who are unable to articulate symptoms, alerting parents to impending hypoglycaemia with predictive alerts. Furthermore, the Dexcom Share feature allows for parents to monitor glucose levels, allowing them to intervene within a timely fashion. Support and education for parents and carers of children with HH will be required to ensure optimal prevention of hypoglycaemia without burden.

In terms of glycaemia, children using Dexcom G5 had a higher proportion of hypoglycaemia than those reported using the Dexcom G6 cohort. Although this is a smaller cohort (n=5 only), these children, aged 0 to 2 years old, also had identified genetic mutations rendering them at confirmed risk of hypoglycaemia. We note children also spend a degree of time above range (>10mmol/L; >180mg/dL), in contrast to the normal range data for children without diabetes (26), which may reflect overcorrection of hypoglycaemia. This may also explain the relatively effective treatment and low frequency of hypoglycaemia (percentage time <63mg/dL; <3.5mmol/L was 1.0%).

The major strength of the study is the population. We recruited a group of children across a wide age range with the rare and complex condition of HH in a tertiary setting. Additionally, the volume of paired data points collected are the largest and acceptable for an accuracy study, and included both hospital and home environment glucose profiles, ensuring the results are applicable to in-patient and out-patient monitoring. Furthermore, a significant proportion of paired data points were in the hypoglycaemic range, demonstrating CGM accuracy in the most clinically important range.

The main limitation of the study is missing quality of life data for some participants. Despite this, our data demonstrate a significant improvement in family impact, parent-reported quality of life, and child-reported quality of life up to the age of 13years old. These findings are in keeping with the significant morbidity, psychosocial and financial burden for children and families affected with HH (27, 28). Data were only available for two participants in the 13 to 16 years old age group.

Finally, this dataset provides further evidence for access to CGM to be considered and widened for people with recurrent hypoglycaemia due to HH. Furthermore, these results could assist with other diseases, including those with neonatal and paediatric diabetes, cystic fibrosis and glycogen storage disorders, but further research in these areas is needed. The clinical effectiveness in reducing exposure to hypoglycaemia compared to usual care can now be assessed and, critically, cost effectiveness of CGM in HH can be evaluated (29). The potential to prevent hospital admissions and reduce length of stay, along with longer-term impact of reducing disability and the direct and indirect costs associated should be considered. Future studies incorporating artificial intelligence and machine learning algorithms may further reduce the burden of hypoglycaemia (30).

## Conclusion

Data from Dexcom G5 and G6 CGM systems accurately reflect CBG in HH, including at the time of hypoglycaemia. Accuracy combined with significant improvements in quality of life for children and their parents suggests that the use of CGM should be considered as a standard of care for children with HH.

## Data availability statement

Data cannot be shared publicly because it is data from the National Health Service. Requests to access the datasets should be directed to nick.oliver@imperial.ac.uk and pratik.shah6@nhs.net.

## Ethics statement

Ethical approval was granted by the London Fulham NHS Research and Ethics Committee. The studies were conducted in accordance with the local legislation and institutional requirements. Written informed consent for participation in this study was provided by the participants’ legal guardians/next of kin.

## Author contributions

MS: Data curation, Formal Analysis, Investigation, Methodology, Project administration, Writing – original draft, Writing – review & editing. PA: Formal Analysis, Writing – original draft, Writing – review & editing. CG: Data curation, Investigation, Methodology, Project administration, Writing – review & editing. LD: Data curation, Investigation, Methodology, Project administration, Writing – review & editing. KM: Data curation, Investigation, Methodology, Project administration, Writing – review & editing. NO: Conceptualization, Data curation, Formal Analysis, Funding acquisition, Investigation, Methodology, Project administration, Writing – original draft, Writing – review & editing. PS: Conceptualization, Data curation, Formal Analysis, Funding acquisition, Investigation, Methodology, Project administration, Writing – original draft, Writing – review & editing.

## References

[1] Hussain K Blankenstein O De Lonlay P Christesen HT. Hyperinsulinaemic hypoglycaemia: biochemical basis and the importance of maintaining normoglycaemia during management. Arch Dis Child. (2007) 92(7):568–70. doi: 10.1136/ADC.2006.115543 PMC2083756 17588969

[2] Helleskov A Melikyan M Globa E Shcherderkina I Poertner F Larsen AM. Both low blood glucose and insufficient treatment confer risk of neurodevelopmental impairment in congenital hyperinsulinism: A multinational cohort study. Front Endocrinol (Lausanne) (2017) 8:156. doi: 10.3389/FENDO.2017.00156 28740482 PMC5502348

[3] Burns CM Rutherford MA Boardman JP Cowan FM. Patterns of cerebral injury and neurodevelopmental outcomes after symptomatic neonatal hypoglycemia. Pediatrics (2008) 122(1):65–74. doi: 10.1542/PEDS.2007-2822 18595988

[4] Barnhardt C Borsting E Deland P Pham NP Vu T. Relationship between visual-motor integration and spatial organization of written language and math. Optom Vis Sci (2005) 82(2):138–42. doi: 10.1097/01.OPX.0000153266.50875.53 15711461

[5] Shah R Harding J Brown J Mckinlay C. Neonatal glycaemia and neurodevelopmental outcomes: A systematic review and meta-analysis. Neonatology (2019) 115(2):116–26. doi: 10.1159/000492859 30408811

[6] Ludwig A Enke S Heindorf J Empting S Meissner T Mohnike K. Formal neurocognitive testing in 60 patients with congenital hyperinsulinism. Horm Res Paediatr (2018) 89(1):1–6. doi: 10.1159/000481774 29151084

[7] Shah P Rahman SA Demirbilek H Güemes M Hussain K. Hyperinsulinaemic hypoglycaemia in children and adults. Lancet Diabetes Endocrinol (2017) 5(9):729–42. doi: 10.1016/S2213-8587(16)30323-0 27915035

[8] Avari P Reddy M Oliver N. Is it possible to constantly and accurately monitor blood sugar levels, in people with Type 1 diabetes, with a discrete device (non-invasive or invasive)? Diabetes Med (2020) 37(4):532–44. doi: 10.1111/dme.13942 30803028

[9] Battelino T Conget I Olsen B Schütz-Fuhrmann I Hommel E Hoogma R. The use and efficacy of continuous glucose monitoring in type 1 diabetes treated with insulin pump therapy: a randomised controlled trial. Diabetologia (2012) 55(12):3155–62. doi: 10.1007/s00125-012-2708-9 PMC3483098 22965294

[10] Type 1 diabetes in adults: diagnosis and management, Guidance and guidelines (NG17). NICE (2015). Available at: https://www.nice.org.uk/guidance/ng17/chapter/1-Recommendations#blood-glucose-management-2.

[11] Martino M Sartorelli J Gragnaniello V Burlina A. Congenital hyperinsulinism in clinical practice: From biochemical pathophysiology to new monitoring techniques. Front Pediatr (2022) 10:901338. doi: 10.3389/FPED.2022.901338 36210928 PMC9538154

[12] Win M Beckett R Thomson L Thankamony A Beardsall K. Continuous glucose monitoring in the management of neonates with persistent hypoglycemia and congenital hyperinsulinism. J Clin Endocrinol Metab (2022) 107(1):E246–53. doi: 10.1210/CLINEM/DGAB601 PMC8830056 34407200

[13] Rayannavar A Elci OU Mitteer L De León DD. Continuous glucose monitoring systems: Are they useful for evaluating glycemic control in children with hyperinsulinism? Horm Res Paediatr (2019) 92(5):319–27. doi: 10.1159/000506230 PMC7192768 32208390

[14] Worth C Dunne MJ Salomon-Estebanez M Harper S Nutter PW Dastamani A. The hypoglycaemia error grid: A UK-wide consensus on CGM accuracy assessment in hyperinsulinism. Front Endocrinol (Lausanne) (2022) 13:1016072. doi: 10.3389/FENDO.2022.1016072 36407313 PMC9666389

[15] Alsaffar H Turner L Yung Z Didi M Senniappan S. Continuous Flash Glucose Monitoring in children with Congenital Hyperinsulinism; first report on accuracy and patient experience. Int J Pediatr Endocrinol (2018) 2018(1):3. doi: 10.1186/S13633-018-0057-2 29599801 PMC5870486

[16] Worth C Tropeano Y Gokul PR Cosgrove KE Salomon-Estebanez M Senniappan S. Insight into hypoglycemia frequency in congenital hyperinsulinism: evaluation of a large UK CGM dataset. BMJ Open Diabetes Res Care (2022) 10(3):e002849. doi: 10.1136/BMJDRC-2022-002849 PMC9185472 35675953

[17] PedsQL TM. Available at: https://www.pedsql.org/ (Accessed August 9, 2022).

[18] Clarke W Kovatchev B. Statistical tools to analyze continuous glucose monitor data. Diabetes Technol Ther (2009) 11(S1):S–45-S-54. doi: 10.1089/dia.2008.0138 PMC2903980 19469677

[19] Moscardó V Giménez M Oliver N Hill NR. Updated software for automated assessment of glucose variability and quality of glycemic control in diabetes. Diabetes Technol Ther (2020) 22(10):701–8. doi: 10.1089/dia.2019.0416 PMC7591379 32195607

[20] Clarke W Cox D Gonder-Frederick L Carter W Pohl S. Evaluating clinical accuracy of systems for self-monitoring of blood glucose. Diabetes Care (1987) 10(5):622–8. doi: 10.2337/DIACARE.10.5.622 3677983

[21] Battelino T Phillip M Bratina N Nimri R Oskarsson P Bolinder J. Effect of continuous glucose monitoring on hypoglycemia in type 1 diabetes. Diabetes Care (2011) 34(4):795–800. doi: 10.2337/DC10-1989 21335621 PMC3064030

[22] Szadkowska A Czyżewska K Pietrzak I Mianowska B Jarosz-Chobot P Myśliwiec M. Hypoglycaemia unawareness in patients with type 1 diabetes. Pediatr Endocrinol Diabetes Metab (2018) 2018(3):126–34. doi: 10.5114/PEDM.2018.80994 30786677

[23] Heinemann L Freckmann G Ehrmann D Faber-Heinemann G Guerra S Waldenmaier D. Real-time continuous glucose monitoring in adults with type 1 diabetes and impaired hypoglycaemia awareness or severe hypoglycaemia treated with multiple daily insulin injections (HypoDE): a multicentre, randomised controlled trial. Lancet (2018) 391(10128):1367–77. doi: 10.1016/s0140-6736(18)30297-6 29459019

[24] Reddy M Jugnee N El Laboudi A Spanudakis E Anantharaja S Oliver N. A randomized controlled pilot study of continuous glucose monitoring and flash glucose monitoring in people with Type 1 diabetes and impaired awareness of hypoglycaemia. Diabetes Med (2018) 35(4):483–90. doi: 10.1111/dme.13561 PMC5888121 29230878

[25] Charleer S De Block C Nobels F Radermecker RP Lowyck I Mullens A. Sustained impact of real-time continuous glucose monitoring in adults with type 1 diabetes on insulin pump therapy: Results after the 24-month RESCUE study. Diabetes Care (2020) 43(12):3016–23. doi: 10.2337/DC20-1531 33067260

[26] Dubose SN Kanapka LG Bradfield B Sooy M Beck RW Steck AK. Continuous glucose monitoring profiles in healthy, nondiabetic young children. J Endocr Soc (2022) 6(6):bvac060. doi: 10.1210/JENDSO/BVAC060 35506147 PMC9049110

[27] Banerjee I Raskin J Arnoux JB De Leon DD Weinzimer SA Hammer M. Congenital hyperinsulinism in infancy and childhood: Challenges, unmet needs and the perspective of patients and families. Orphanet J Rare Dis (2022) 17(1):61. doi: 10.1186/S13023-022-02214-Y 35183224 PMC8858501

[28] Auckburally SH Worth C Salomon-Estebanez M. Families’ Experiences of continuous glucose monitoring in the management of congenital hyperinsulinism: A thematic analysis. Front Endocrinol (Lausanne) (2022) 13:894559. doi: 10.3389/FENDO.2022.894559 35928891 PMC9343578

[29] Ng SM Dearman S Fisher M Mushtaq T Randell T. Case for funding of continuous glucose monitoring systems for patients with recurrent hypoglycaemia. Arch Dis Child (2022) 6:archdischild–2022–323872. doi: 10.1136/ARCHDISCHILD-2022-323872 36202595

[30] Worth C Nutter PW Dunne MJ Salomon-Estebanez M Banerjee I Harper S. HYPO-CHEAT’s aggregated weekly visualisations of risk reduce real world hypoglycaemia. Digit Heal (2022) 8:205520762211297. doi: 10.1177/20552076221129712 PMC9580093 36276186

