# Supplementary material for: Accuracy and impact on quality of life of real-time continuous glucose monitoring in children with hyperinsulinaemic hypoglycaemia
Source: Front Endocrinol (Lausanne). 2023 Sep 26;14:1265076. doi: 10.3389/fendo.2023.1265076 (PMC10562688; doi:10.3389/fendo.2023.1265076)
Supplement: Supplementary file 1 [file DataSheet_1.docx]

**Supplementary Material**

**Figure 1: Clarke error grids and Bland Altmann analysis for Dexcom G6 (A and B) and G5 data (C and D) compared with reference capillary blood glucose.** The overall mean bias for Dexcom G6 was +2.0 mg/dL (+0.1mmol/L) and 95% limits of agreement were -37.2mg/dL (-2.1mmol/L), +33.2 mg/dL (+1.8mmol/L). The overall mean bias for Dexcom G5 was -1.3 mg/dL (-0.07mmol/L) and 95% limits of agreement were -33.5mg/dL (-1.9mmol/L), +31.0 mg/dL (+1.7mmol/L).
